# Molecular Modeling-Based Evaluation of hTLR10 and Identification of Potential Ligands in Toll-Like Receptor Signaling

**DOI:** 10.1371/journal.pone.0012713

**Published:** 2010-09-16

**Authors:** Rajiv Gandhi Govindaraj, Balachandran Manavalan, Gwang Lee, Sangdun Choi

**Affiliations:** 1 Department of Molecular Science and Technology, Ajou University, Suwon, Korea; 2 Institute for Medical Sciences, Ajou University School of Medicine, Suwon, Korea; Keio University, Japan

## Abstract

Toll-like receptors (TLRs) are pattern recognition receptors that recognize pathogens based on distinct molecular signatures. The human (h)TLR1, 2, 6 and 10 belong to the hTLR1 subfamilies, which are localized in the extracellular regions and activated in response to diverse ligand molecules. Due to the unavailability of the hTLR10 crystal structure, the understanding of its homo and heterodimerization with hTLR2 and hTLR1 and the ligand responsible for its activation is limited. To improve our understanding of the TLR10 receptor-ligand interaction, we used homology modeling to construct a three dimensional (3D) structure of hTLR10 and refined the model through molecular dynamics (MD) simulations. We utilized the optimized structures for the molecular docking in order to identify the potential site of interactions between the homo and heterodimer (hTLR10/2 and hTLR10/1). The docked complexes were then used for interaction with ligands (Pam_3_CSK_4_ and PamCysPamSK_4_) using MOE-Dock and ASEDock. Our docking studies have shown the binding orientations of hTLR10 heterodimer to be similar with other TLR2 family members. However, the binding orientation of hTLR10 homodimer is different from the heterodimer due to the presence of negative charged surfaces at the LRR11-14, thereby providing a specific cavity for ligand binding. Moreover, the multiple protein-ligand docking approach revealed that Pam_3_CSK_4_ might be the ligand for the hTLR10/2 complex and PamCysPamSK_4,_ a di-acylated peptide, might activate hTLR10/1 hetero and hTLR10 homodimer. Therefore, the current modeled complexes can be a useful tool for further experimental studies on TLR biology.

## Introduction

Toll-like receptors (TLRs) are evolutionarily conserved membrane-bound pattern recognition receptors (PRRs) that recognize a broad spectrum of microbial components such as lipopeptides and non-self nucleic acids [1]. TLRs play a central role in innate immunity and are required for the development of adaptive immune responses. TLRs are type I transmembrane glycoproteins that consist of an extracellular domain/ectodomain (ECD), a single transmembrane spanning segment and a globular cytoplasmic Toll/interleukin (IL)-1 receptor (TIR) domain [2], [3]. The ECDs encompass 19–25 tandem copies of a motif known as the leucine-rich repeat (LRR), whose primary function appears to be ligand recognition and the formation of protein-protein interactions with its partners [4], [5], [6]. The cytoplasmic TIR domain recruits the adaptor protein that is essential for the downstream signaling. To date, 10 hTLRs (human TLRs) and 12 mTLRs (mouse TLRs) have been identified, each of which responds to specific microbial products [2], [7], [8]. TLRs are categorized into six major families: TLR1, TLR3, TLR4, TLR5, TLR7 and TLR11. The classification is mainly based on amino acid similarities and the ligand properties [9]. The present study mainly focused on the TLR1 family, which includes TLR1, TLR2, TLR6 and TLR10.

Although TLR10 has been identified in humans and its equivalent homologs can be found in other mammals, it is absent from mice due to a retrovirus insertion. TLR10 is predominantly expressed in immune cell-rich tissues, such as the small intestine, stomach, thymus, peripheral blood lymphocytes, lymphnodes and tonsils [10], [11]. Co-immunoprecipitation studies have shown the self association of TLR10, as well as TLR1 and TLR2 via the extracellular domain of these receptors, which plays a pivotal role in inducing type I interferon in plasmacytoid dendritic cells (PDC) [12]. The ligand specificity has been elucidated for most of the TLRs. Specifically, TLR2 and TLR4 recognize Gram-positive and Gram-negative bacterial cell wall products, respectively, while TLR5 recognizes a structural epitope of bacterial flagellin and TLR3, 7, 8 and 9 have been demonstrated to recognize different forms of microbial-derived nucleic acids [2], [13]. However, the ligand specificity of TLR10 remains elusive. The present study was conducted to identify the hTLR10 self dimerization region as well as its partners and possible ligands to activate these complexes.

Computational modeling has become an essential tool in guiding and enabling rational decisions with respect to hypothesis driven biological research. In the absence of an experimentally determined structure, homology modeling could provide a rational opportunity to obtain a reasonable 3D structure. The goal of homology modeling is to model or predict the structural coordinates of a query protein based on the known structure of a sequence homology (template). The 3D structure of a protein provides important information for understanding its biochemical function and interaction properties in molecular detail. Here, we used a combination of homology modeling and MD simulation to construct a detailed 3D model of hTLR10. This model was then used to investigate the nature of the three different binding modes with hTLR1 and hTLR2, which provided the complex formation of the hTLR10/1, hTLR10/2 hetero and hTLR10 homodimer. These complex structures were then studied to analyze the intermolecular contacts and to identify the specific residual level of interactions between the two proteins. The results presented in this study demonstrated that the binding orientations are similar among all TLRs; however, the residual interactions with their partners are specific. The hTLR10 complexes were then used for protein-ligand docking to identify the potential ligands that activate the TLR10 signaling. Finally, we have provided possible ligands for the hTLR10 complex activation.

## Methods

### Homology modeling

The primary sequence of hTLR10 (accession number: AAQ88667) and crystal structure coordinates of hTLR1 (PDB ID: 2Z7X-B) were loaded into the Molecular Operating Environment (MOE). The primary structure of hTLR1 and hTLR10 were aligned and carefully checked to avoid deletions or insertions in each LRR and then corrected manually based on the LRR motif identified by TollML [14], [15]. A series of 10 hTLR10 models were independently constructed with MOE by using the Boltzmann-weighted randomized procedure. There was no difference in the number and organization of secondary structural elements and no significant main chain deviation among the 10 models developed. However, the model with the highest packing score was selected for full energy minimization (MOE packing score is −2.5478). The selected final model was subjected to MD simulation for structural refinement [16]. The glycosylation sites present in the final model were predicted by using the NetOglyc server [17].

### Molecular dynamics simulation

The MD simulations were conducted using the GROMACS 3.3.3 software [18]. The individual structures of the modeled hTLR10, 1 and 2 were placed into a cubic box maintaining 10 Å between the box edges and the protein surface. The resulting system was then solvated with the simple point charge (SPC) water molecule and then minimized by using steepest descent method with GROMOS96 43a1 force field. Periodic boundary conditions were applied in all directions and the system was neutralized by adding appropriate counter ions (Na^+^ or Cl^−^). A twin range cutoff was used for long-range interaction: 9 Å for van der Waals interactions and 14 Å for electrostatic interactions. All bond lengths were constrained with the LINCS algorithm [19]. The SETTLE algorithm was applied to constrain the geometry of water molecules [20]. The energy minimized system was subjected to 100 ps equilibration and subsequently used in the 2 ns production with a time step of 1 femtosecond (fs) at constant temperature (300 K), pressure (1 atm) and number of particles, without any position restraints. In each case, the final conformation obtained at the end of the simulation was further refined by energy minimization for molecular docking. The refined model was validated using the PROCHECK program [21] and the Verify 3-dimensional (3D) server [22], [23].

### Molecular docking

The three widely accepted rigid-body protein-protein docking programs, GRAMM-X [24], ZDOCK [25] and RosettaDock [26] were used to predict and assess the interactions between hTLR10/1, hTLR10/2 and hTLR10 homodimer complexes. Each docking method returned the 100 most probable predictions out of thousands of candidates based on geometry, hydrophobicity and electrostatic complementarity of the molecular surface. We thus performed restrained pairwise docking for hTLR10 heterodimer complex based on the comparative studies results. Our comparative studies clearly indicated that C-terminal region of LRR11–14 plays a crucial role in dimerization. When we used our comparative studies results as a constraint for homodimer, we obtained the complex similar to heterodimer. However, the active site was positioned far away from the ligand binding region and hence, it was not appropriate to conduct further studies. Consequently, the residues located near the hTLR10 active site were considered as a constraint in our docking studies. The buried surface interaction area of dimer models were calculated using the PROTORP server (protein-protein interface analysis) [27].

### Electrostatic potential calculation

The electrostatic potential surface of the hTLR10, 2 and 1 were calculated using the PyMOL APBS (http://apbs.sourceforge.net) tools. The molecular surfaces and ribbons of the figures were built and colored with the PyMOL visualizing tool [28].

### Computational alanine scanning to predict the binding energy Hot-spots in the complex interface

The hTLR10/1, hTLR10/2 hetero and hTLR10 homodimer were submitted to the Robetta Alanine scanning server (http://robetta.bakerlab.org/alascansubmit.jsp) to predict the energetically important amino acid residues in its interface. The alanine scanning server calculates the effects of alanine mutations on the binding free energy of a protein-protein complex by using a simple free energy function. The program replaces each of the interface residues with alanine residue and computes the effect of this mutation on the binding free energy of the complex. This analysis serves as the identification of important residues that are crucial for hTLR10 homo and heterodimer formation.

### Construction and geometry optimization of hTLR10 ligands

Two ligands, Pam_3_CSK_4_ and PamCysPamSK_4_, have been predicted to activate the hTLR10 complexes. The crystal structure of hTLR1/2/Pam_3_CSK_4_ complex is known, and we took the Pam_3_CSK_4_ site for docking. In case of PamCysPamSK_4_, we modified the Pam_3_CSK_4_ at C2 position. Partial charges were added to protonated and unprotonated molecules using the Merck Molecular Force Field 94X (MMF94X), which is suitable for small molecules [29]. All structures were energy minimized using the conjugated gradient/truncated Newton optimization algorithm with a convergence criterion of 0.05 kcal/mol, ε = 1.

### Binding site selection and exploration

The Site Finder module of MOE 2008.10 was used to identify the possible substrate binding pockets within the newly generated 3D structure of the hTLR10/1, hTLR10/2 hetero and hTLR10 homodimers. Hydrophobic or hydrophilic alpha spheres served as probes denoting zones of tight atom packing. The alpha spheres were used as centroids for the creation of dummy atoms to define the potential sites throughout the docking process.

### hTLR10 complex-ligand docking

Protein-ligand docking was conducted using MOE-Dock 2008.10 and ASEDock. The docking procedure has been previously described [30], and the same procedure was followed for hTLR10 complexes and its ligands.

### Validation of the hTLR10 ligand docking process via hTLR1/2/Pam_3_CSK_4_ docking

Two docking methods, MOE-Dock 2008.10 and ASEDock, were used to assess the validity of the hTLR10 complex-ligand docking predictions by calculating possible bound conformations of hTLR1/2/Pam_3_CSK_4_complexes. The crystal structure of hTLR1/2 was retrieved from the PDB and prepared for docking, and partial charges and hydrogen atoms were added. The obtained docking poses (hTLR1/2/Pam_3_CSK_4_) were then compared with the original crystal structure.

## Results and Discussion

### Molecular model of hTLR10 and evaluation

**Figure 1:**
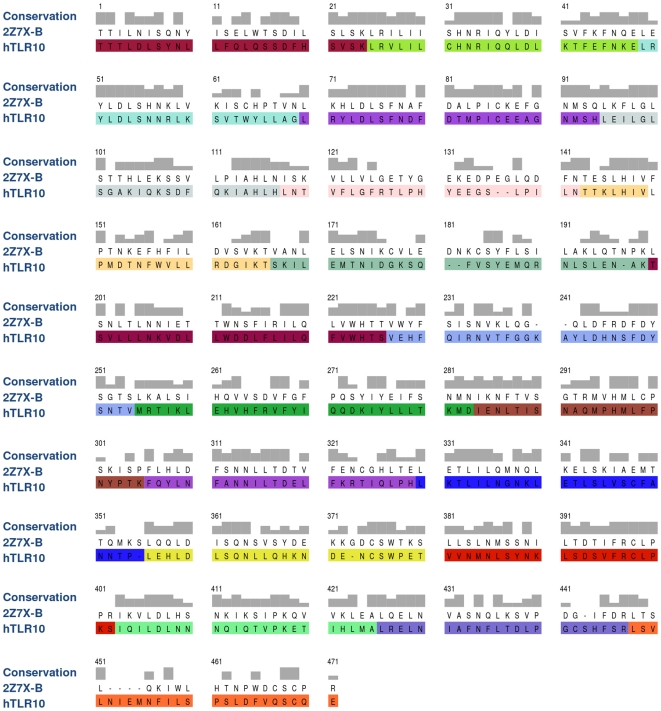
Sequence alignment used to build hTLR10, based on the hTLR1 template retrieved by MOE. Gray blocks: level of sequence similarity. Tallest blocks: identical residues at that position. Intermediate blocks: non-identical residues that are relatively conservative with respect to their properties. Small blocks: residues sharing mild conservation with respect to structure or function. The absence of a block indicates no appreciable structure/function conservation. Gaps in one sequence relative to the other are indicated by dashes. 2Z7X-B denotes hTLR1. The nineteen LRR are highlighted and contrasted by varying the colors.

We built the 3D structure of hTLR10 using the crystal structure of hTLR1, which serves as a suitable template (PDB ID 2Z7X-B) [31]. Generally, TLR ectodomains are composed of a repeated number of LRR, which vary in numbers among the known TLRs [32]. Recent modeling studies of endosomal TLRs (hTLR7, 8, 9) have shown that researchers used multiple templates to derive an optimum alignment for all endosomal LRRs [33]. Conversely, we used a single template (2Z7X-B) because it has 19 LRR, which are aligned well with the 19 LRR in the target hTLR10. The primary sequence alignment of hTLR10 and its template was used to construct the final model (Figure 1). The identity and similarity between the target and template were 40.74% and 59.26%, respectively. Additionally, the sequence identity of each LRR between the target/template is listed in Table 1. Direct comparison of the modeled hTLR10 with its template showed that the structural differences observed in the loop regions of the LRR6, LRR14, LRR15, LRR19 and the rest of the regions were similar to that of the template with RMSD values of 1.14 Å. These structural differences occur away from the ligand binding sites (Figure S1). Moreover, when we compared hTLR10 with the available crystal structure of hTLR2 (in the same family) [31], we found that the variation in loop regions in LRR1, LRR2, LRR3, LRR4, LRR5, LRR7, LRR8, LRR11, and the rest of the regions was similar to that of hTLR2 with RMSD values of 1.54 Å. The final 3D structure of hTLR10 showed large characteristic “horseshoe-like” structures with concave surfaces formed by 19 parallel β-strands, whereas the convex surface contains more diverse secondary structural elements, including a variety of different length loops, 11 α helices and three 3_10_ helices. Like all TLRs, hTLR10 also contains the *N*-linked glycosylation consensus sites. The glycans were exemplified to be non-functional for ligand binding [25], [28]–[31]. Hence, we predicted seven glycosylation sites for hTLR10, which are located in the LRR4 (Asn91), LRR6 (Asn142), LRR8 (Asn189), LRR10 (Asn231), LRR12 (Asn254), LRR15 (Asn369) and LRR16 (Asn378). However, our prediction also revealed that none of the glycosylation sites contributed in the dimerization and ligand binding sites. The predicted glycosylation sites are similar to those of TLR1 family members [34].

**Table 1:** Sequence identities (%) of target-template of each LRR pairs.

LRRs	hTLR10	LRRs	hTLR10
1	45.83	11	24.14
2	62.5	12	40.91
3	38.1	13	41.67
4	68	14	36
5	47.83	15	37.5
6	26.09	16	45.45
7	41.67	17	43.48
8	23.33	18	36.36
9	44.44	19	20.83
10	50	Avg	40.74

Note: LRRs, Leucine rich repeats; hTLR10, human Toll like receptor10; Avg, average value.

### Structure refinement and stability evaluation by molecular dynamics

**Figure 2:**
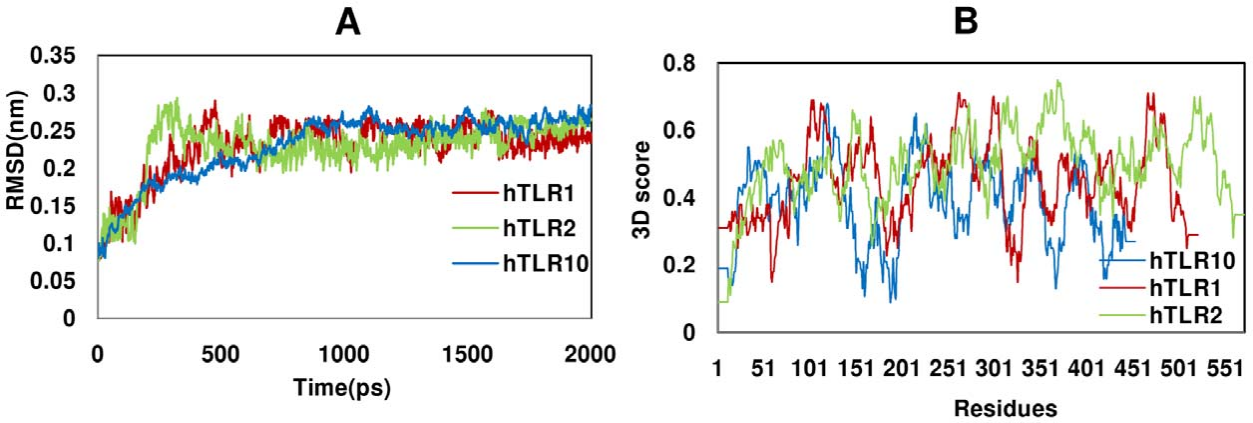
Structure refinement and stability evaluation. (**A**) The MD trajectory-based analyses for the model refinement. The RMSD plot of hTLR10, 2 and 1 during the dynamic simulation. (**B**) The 3D structure profile plot for hTLR10, 2 and 1. The refined hTLR1 and hTLR10 are shown in blue and red, whereas hTLR2 is shown in green.

We performed 2 ns MD simulations to explore the structural stability of the model structure of hTLR10 along with the crystal coordinates of hTLR2 and 1. The crystal structures of the hTLR2/1 heterodimer along with its ligand were solved at 1.8 Å resolutions [31]. The ligand was removed from the heterodimer and the dimer was separated into hTLR1 and hTLR2. To evaluate the overall stability of these models, we calculated the RMSD from the initial structure of all backbone Cα atoms as a function of simulation time, as shown in Figure 2A. The hTLR1 and hTLR2 reached equilibrium around 500 ps, whereas hTLR10 reached the plateau after 900 ps. The RMSD value of the Cα atom between the initial snapshot and the final structure was around 2.8 Å for hTLR10, 2.6 Å for hTLR2 and 2.4 Å for hTLR1, which suggests that the protein remains stable after reaching the equilibrium. The final snapshot at the end of the simulation was selected from the 2 ns and subjected to further energy minimization for fine refinement. The optimized structures of the hTLR10, hTLR2 and hTLR1 were validated using the PROCHECK program and Verify 3D server. Analysis of hTLR10 revealed that 94% of the dihedral angles are found in most favorable regions of the Ramachandran plot and that the remaining 6% are found within the allowed regions (Figure S2). This highlights the excellent geometry of the model. Verify3D reports no values below 0.09, further indicating that all the residues are located in favorable structural environments (Figure 2B). It should be noted that before the simulation, hTLR1 and 2 had a few residues in the outlier region. However, evaluation of the optimized structure of hTLR1 from the MD simulation revealed that 96.1% of the residues were present in the most favorable regions and remaining 3.9% residues were located in allowed regions (Figure S2). Verify3D reported no values below 0.15 (Figure 2B). In the case of hTLR2, 94.14% of the residues were located in most favorable region and 5.85% residues were located in the allowed region (Figure S2). Verify3D reported no values below 0.17 (Figure 2B). This analysis indicates excellent geometry of the crystal structure after the MD simulation and is thus considered a reliable source for further studies.

### Comparison of hTLR10 with hTLR1 and hTLR2

**Figure 3:**
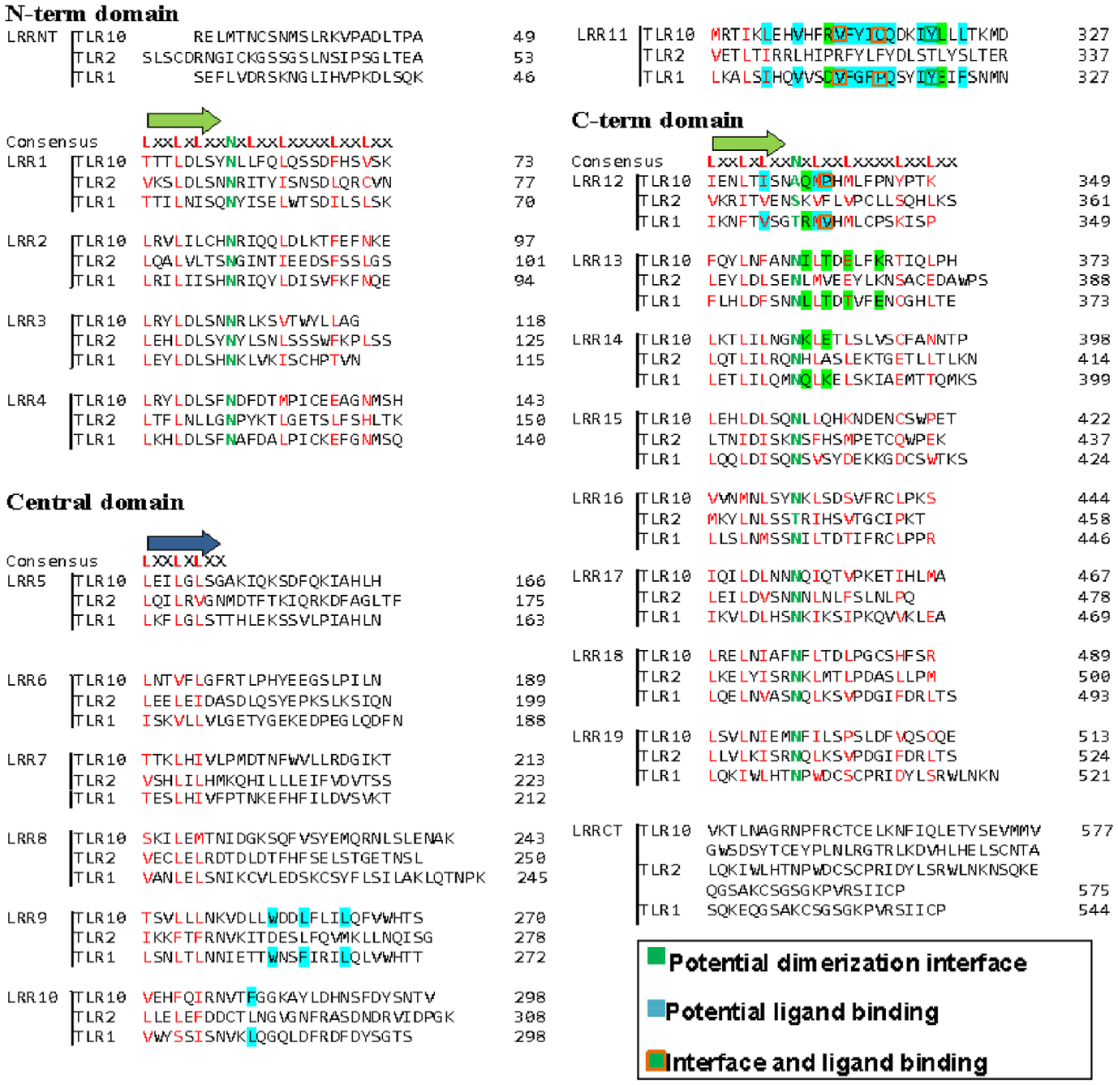
Structure-based sequence alignments of hTLR10, 2 and 1. The hTLR10, 2 and 1 sequences are aligned based on their structures. Conserved leucines and asparagine ladder are written in red and green, respectively. The positions of β-strands are shown above the consensus patterns.

As shown in Figure 3, the structure based sequence alignment of hTLR10 with hTLR2 and 1 shows that the LRR consensus sequence motif “LxxLxLxxNxLxxLxxxxLxxLxx” aligned well at the N-terminal, C-terminal and Central domain. In the above sequence motif, x denotes any amino acids, and L represents leucine, which can be replaced by other hydrophobic amino acids such as isoleucine, valine and phenylalanine. The consensus asparagine (N) in the LRR motif plays an important role in the formation of the hydrogen bonds with the carbonyl backbone of the neighboring strands [5], [35]. The hTLR10 N-terminal domain was composed of 1–4 LRR modules, with each LRR module length being around 24 residues and the structurally important asparagine ladder being conserved. Conversely, the central domain is composed of 5–11 LRR modules and differs considerably from the standard structure, with the lengths of their LRR modules ranging from 20–30 amino acids and their β-sheet conformations deviating significantly from those of the standard LRRs. The absence of an asparagine ladder in the central domain leads to the formation of an unusual β-sheet conformation. However, we observed such unusual regions in hTLR1 and 2. These unusual regions of hTLR1 and 2 possess hydrophobic pockets, which have been reported to play an essential role in ligand recognition [31]. This analysis shows that residues situated in the ligand binding hydrophobic pockets in hTLR2 and 1 are conserved in hTLR10 (Figure 3 shaded by cyan). Hence, we believe that this region in hTLR10 might play a role in ligand recognition. Finally, the C-terminal domain of hTLR10 contains 12–19 LRR modules and the asparagine ladder is conserved in all LRRs, with each LRR module ranging in length from 20–30 amino acids. Bell et al. predicted that there were insertions that occur at the position of LRR12 in hTLR2, LRR4 in hTLR10, and LRR12 in hTLR1 [32]. However, our structure based analysis of hTLR10 along with the crystal structures of hTLR1 and hTLR2 did not reveal such an insertion in these three receptors.

### Receptor dimerization interface

The signaling mechanism of all TLRs is likely involved in dimerization of the ectodomains [36]. However, this can be achieved in various ways by using different receptors and stimulation. It should be noted that the ligand induced dimerization takes place in the TLR signaling, and a few crystal structures have already been solved based on this idea [31], [37], [38]. However, this cannot be conducted computationally; hence, we initially focused on the dimerization mechanism of hTLR10 with hTLR2 and 1 using protein-protein docking. The procedure of protein-protein docking is highly computationally oriented. The reliability of docking results strongly depends on the quality of the docking methods. To verify the prediction confidence of the TLR-TLR interaction of these three methods, GRAMM-X, ZDOCK and RosettaDock, we restrainedly inputted hTLR1 and hTLR2, for which the heterodimeric crystal structure is known, as test cases [31]. The native dimerization structure of hTLR2/1 was found in the top 100 solutions and it was ranked 23rd by GRAMM-X, 1st by RosettaDock and 80th by Z-DOCK. These results demonstrate that our docking protocol was reliable; therefore, we used it in the subsequent hTLR10/2, hTLR10/1 hetero and hTLR10 homodimer docking calculations.

We conducted restrained rigid-body docking of the hTLR10 homo and heterodimer. Each docking returned the 100 most probable models from unbound monomer components. Thus, each complex received a total of 300 candidate models separated into three sets. Some models from the same set had similar conformations, whereas most differed considerably from one another. There were some shared models (intersection) across both sets for each complex. These shared models were considered as more confident solutions than others. The optimal docking solution was selected for each complex from the 300 candidates based on the following criteria: (i) models that do not exist in the intersection of the three resulting sets were excluded; (ii) only those models in which the dimerization geometry was supported by the results obtained from our comparative studies were included. The comparative studies clearly indicated that the dimerization region is located at the border of the central region followed by the C-terminal end (Figure 3 represented in green color shade). Additionally, we considered the active site region to be located on the adjacent sides in the dimer interface. This two-step filtering led to a unique solution. The ZDOCK/GRAMM-X/RosettaDock ranking and the buried surface interaction area of all optimal models are provided in Table 2.

**Table 2:** Ranking and interaction area of the selected docking models.

Complexes	GRAMM-X	Z-DOCK	RosettaDock	Interaction Area (Å^2^)
hTLR10/hTLR2	33	26	1	945.58
hTLR10/hTLR1	45	73	8	809.63
hTLR10/hTLR10	81	59	13	858.74

### hTLR10-hTLR2 complex

**Figure 4:**
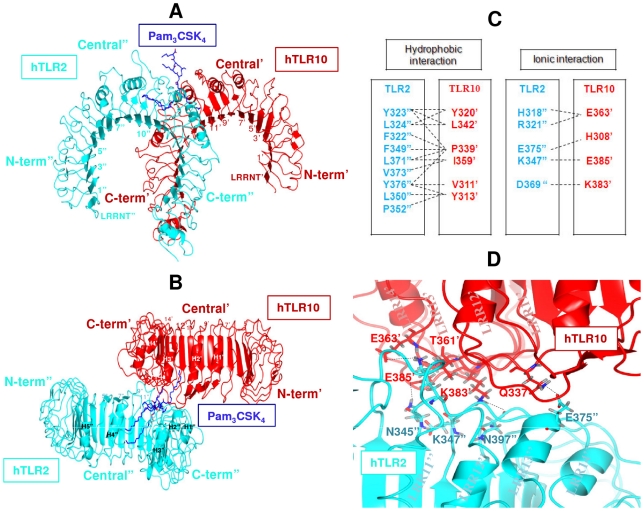
Protein-protein docking model of the hTLR10/2 complex. hTLR2 and hTLR10 are shown schematically in cyan and red, respectively. Each LRR module is numbered and the N-terminal, central, and C-terminal subdomains are labeled. A and B, side view (**A**) and top view (**B**) of the complex in ribbon representation. (**C**) Residues involved in the heterodimer interface in the hTLR10/2 are shown in cyan and red, respectively. The interactions between the residues are depicted by dashed lines. (**D**) Hydrogen bonds between hTLR10 and hTLR2 (cyan) are marked by black dashes.

The orientation and interaction of the final hTLR10/2 docked complex was similar to the hTLR2/1 crystal complex and TLR10/1 (Figure 4A&B). The buried surface at the interface of the hTLR10/2 complex constitutes 945.58 Å^2^ from the hTLR10 and 958.39 Å^2^ from hTLR2, which is in the range of typical physiological interaction surfaces. Based on the solvation energy calculated from the buried surface area and specific electrostatic interaction, the dimer interface was judged by PROTORP server. There are 14 residues from the hTLR10 that make contact (ΔASA>1 Å^2^) with 15 residues from hTLR2. The non-covalent interactions across the hTLR10/2 interface are listed in Figure 4C. The hTLR10/2 interface possesses a small hydrophobic core located in the central region (LRR11) surrounded by hydrogen bonding and ionic interaction in the periphery. Five hydrogen bonds are present in the interface of the hTLR10/2 complex (Figure 4D), which includes a double hydrogen bond between the K347^″^ side chain and the OH group of T361^′^. The carbonyl backbone of N397^″^, carboxylic group of E375^″^ and carboxamide group of N345^″^ form a hydrogen bond with the NH_2_ group of K383^′^, NH2 group of Q337^′^ and carboxylic group of E385^′^, respectively. Single apostrophes are used for the hTLR10 residues and double apostrophes for the hTLR2 to differentiate them from the ligand throughout the article.

### hTLR10-hTLR1 complex

**Figure 5:**
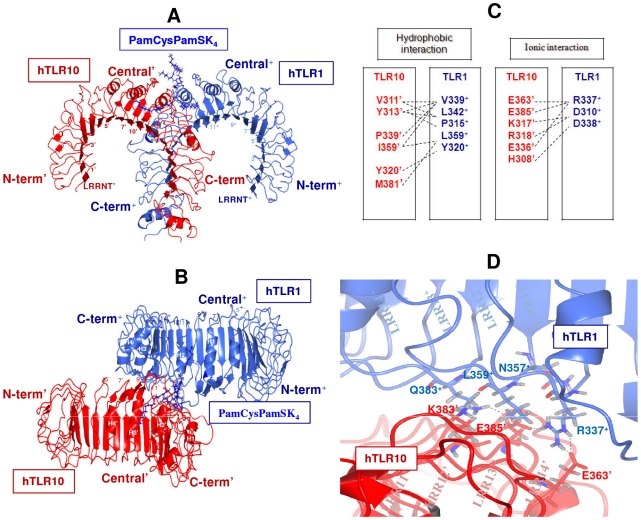
Docking model of the hTLR10/1 complex. The hTLR10 and hTLR1 are shown schematically in red and light blue, respectively. Each LRR module is numbered and the N-terminal, central, and C-terminal subdomains are labeled. A and B, side view (**A**) and top view (**B**) of the complex in ribbon representation. (**C**) Residues involved in the heterodimer interface in hTLR10/1 are shown in red and blue, respectively. The interactions between the residues are depicted by dashed lines. (**D**) hTLR10 residues interact with hTLR1 by making hydrogen bonds (black dashes).

The buried interface surface area between the hTLR10 and the hTLR1 is 809.63 Å^2^ from hTLR10 and 798.12 Å^2^ from hTLR1 (Figure 5A&B). There are 15 residues from hTLR10 that makes contact (ΔASA>1 Å^2^) with 10 residues from hTLR1. The non-covalent interactions such as hydrophobic and ionic interactions across the hTLR10 and the hTLR1 are listed in Figure 5C. Five hydrogen bonds are present in the interface of the hTLR10/1 complex (Figure 5D). The NH_2_ group of R337^+^ forms two hydrogen bonds with carboxylic group of E385^′^ and E363^′^, respectively. The carboxamide group of Q383^+^ forms a hydrogen bond with the carboxylic group of E385^′^ of hTLR10. The side chain of K383^′^ forms two hydrogen bonds with the backbone oxygen group of L359^+^ and the backbone oxygen group of N357^+^. Single apostrophes are used for the hTLR10 residues and double apostrophes for the hTLR2 to differentiate them from the ligand throughout the article.

### hTLR10-hTLR10 dimer

**Figure 6:**
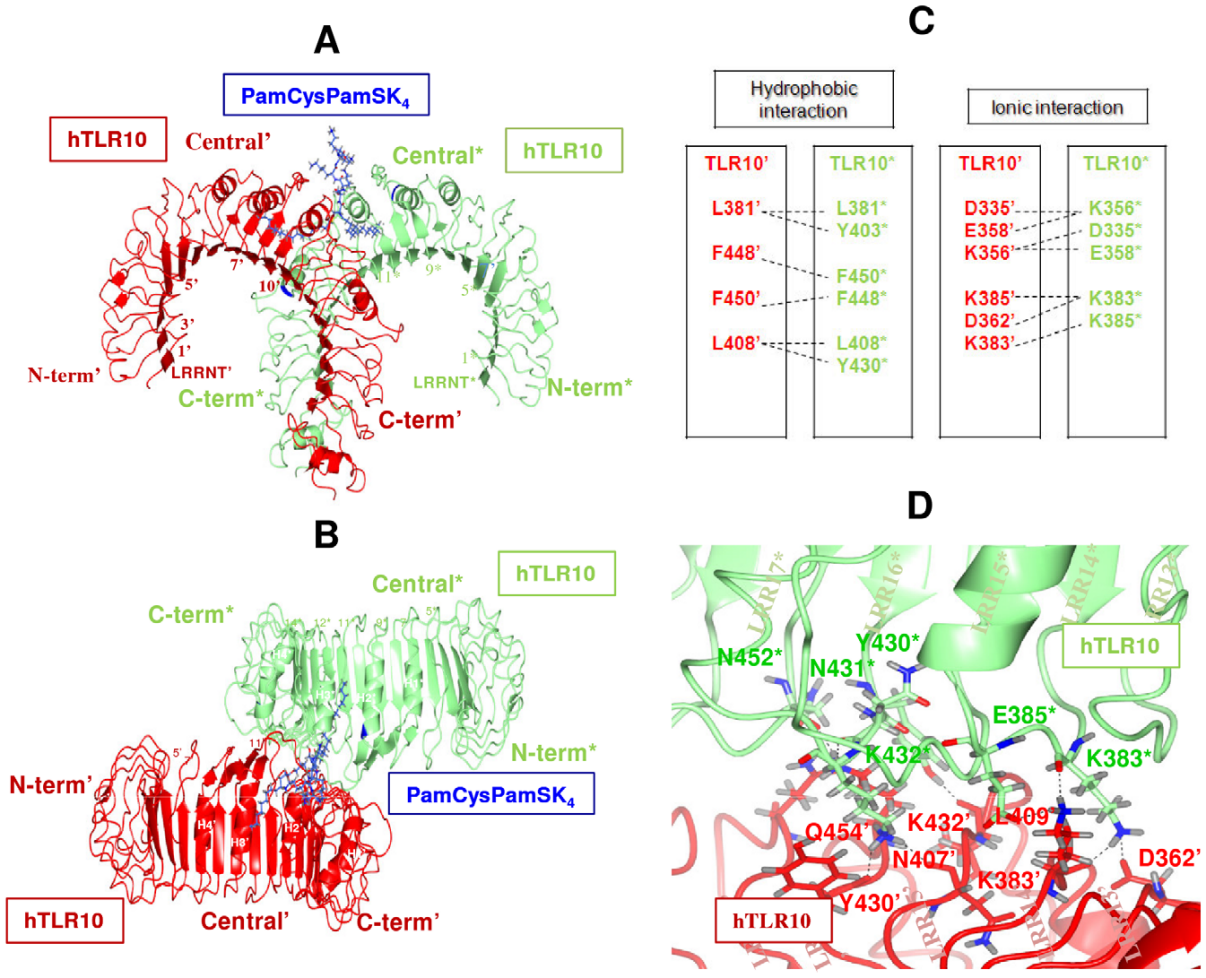
Docking model of the hTLR10/10* complex. hTLR10 and hTLR10* are shown schematically in red and green, respectively. PamCysPamSK_4_ is shown in dark blue. Each LRR module is numbered and the N-terminal, central, and C-terminal subdomains are labeled. A and B, side view (**A**) and top view (**B**) of the complex in ribbon representation. (**C**) Residues involved in the homodimer interface in the hTLR10/10* are shown in red and green, respectively. The interactions between the residues are depicted by dashed lines. (**D**) hTLR10 residues interact with hTLR10 by making hydrogen bonds (black dashes).

The hTLR10 homodimer docking orientation is the same, but the dimerization region is different from the hTLR10 heterodimer complexes. The hTLR10 homodimerization region includes LRR11-17 in comparison to LRR11-14 in the heterodimer complex. Such types of dimerization have been conspicuously noted in TLR4 homodimer. The hTLR10 homodimer docked complex resembles a typical ‘m’ shaped heterodimer, with two N-termini extending outwards in opposite directions and LRRCT modules converging at the center (Figure 6A&B). However, the hTLR10 homodimer possesses more hydrophobic and hydrogen bond interactions than its heterodimer complex. The buried surface area across the hTLR10 homodimer complex is 858.74 Å^2^. There are 13 and 14 residues from hTLR10 chain D and E^*^ that contribute to the above interface. Figure 6C shows the hydrophobic and ionic interaction between the homodimer of hTLR10/10^*^. Eight hydrogen bonds are present at the interface of the hTLR10 homodimer, which includes the carbonyl backbone of N407^′^ and Y430^′^ that forms two hydrogen bonds with the side chain of K432^*^. Similarly, the side chains of K432^′^, Q454^′^, N362^′^ and K383^′^ form hydrogen bonds with the side chains of N404^*^, N425^*^, K356^*^ and E358^*^, respectively. The backbone oxygen group of K409^′^ forms hydrogen bonds with the side chain of Y403^*^ (Figure 6D). Single apostrophes are used for the hTLR10 residues and asterisks for the other hTLR10 monomer to differentiate them from the ligand throughout the article.

The docking results revealed that the amino acid residues across the interface of the hTLR10 homo and heterodimer vary depending on their partners, which clearly demonstrates that hTLR10 binds specifically with its partners. The predicted docked complexes were cross checked by the electrostatic potential of the individual structure. The surface electrostatic calculation from hTLR10 revealed that the highly negative charged surface along with a few positive patches were located at the central region, followed by the C-terminal (LRR11-14) (represented by the green color in Figure 3), whereas hTLR2 and 1 contain highly basic amino acids (Figure 2B). The hTLR10 negatively charged surface (LRR11-14) would bind with hTLR2 and 1 (LRR11-14) based on their respective opposite charges. However, in the case of hTLR10, homodimer formation occurs between LRR11-17 of each monomer. These LRR regions of each monomer have eight negative and four positive charges where only a few residues are involved in the complementary interaction has shown in Figure S3.

### Virtual Ala scanning

An approximate estimation of the individual contributions of the amino acid residues involved in the interaction was obtained by computational methods. We used the Rosetta interface computational mutagenesis approach [39], which is similar in principle to the experimental Ala-scanning mutagenesis procedure, to estimate the change in the binding free energy (ΔΔ*G*_bind_) when each residue at the interface of the hTLR10/2, hTLR10/1 and hTLR10/10* complex is mutated to Ala. We utilized a cut-off of ΔΔ*G*_bind_>1.0kcal/mol to qualitatively identify hot-spot residues that are essential for the interactions. Correctly identified essential residues have predicted and observed (ΔΔ*G*_bind_) values greater than or equal to 1 kcal/mol; whereas correctly identified neutral residues have predicted and observed (ΔΔ*G*_bind_) values less than 1 kcal/mol.

To verify the prediction confidence of alanine-scanning mutagenesis, we input the hTLR2/1 crystal structure as a test case. The program predicted a few residues across the complex interface. Of those, P315 in TLR1 has a value of 2.65. A mutation in this residue leads to loss of the hTLR1/2 signaling [40]. This shows that P315 is essential for the dimerization of hTLR1/2 complex. Therefore, we believe that our protocol is reliable and we subsequently used it to evaluate the docked complexes. The alanine scanning experiments showed that two residues (T361^′^ and K383^′^) from hTLR10 and two residues (K347^″^ and Y376^″^) from hTLR2 were crucial for the formation of hTLR10/2 complex (Table 3), emphasizing their contribution to the complex stability. Conversely, evaluation of the hTLR10/1 complex revealed that two residues from hTLR10 (K383^′^ and E385^′^) and two residues from hTLR1 (R337^+^ and Q383^+^) were indispensible for hTLR10/1 complex formation. Finally, the dimer complex showed that one residue from hTLR10 (K383^′^) and one residue from hTLR10^*^ (K356^*^) were essential for homodimer complex formation. The rest of the residues at both complex interfaces had ΔΔG_bind_ values >0.1 kcal/mol, which suggests that their mutation to Ala does not have any functional consequences on the formation of complexes.

**Table 3:** Results of the predicted contributions of residues through virtual alanine scanning.

TLR10/2			TLR10/1			TLR10/10*		
PDB	Chain	ΔΔG_bind_	PDB	Chain	ΔΔG_bind_	PDB	Chain	ΔΔG_bind_
Y323	A	2.04	Q383	B	1.25	K383	E	1.35
K347	A	2.72	R337	B	1.70	T430	E	3.56
Y376	A	0.92	K383	D	1.04	K383	D	1.32
T361	D	1.23	E385	D	1.01	K432	D	2.68
K383	D	0.93						

Note: Chain A, hTLR2; Chain B, hTLR1; Chain D, hTLR10; Chain E, hTLR10*; PDB, position of mutated residues in the pdb file; ΔΔG_bind_, predicted change in binding free energy upon alanine mutation.

### Possible ligand recognition for hTLR10 based on the cavity volume

Recent studies have revealed the binding of the tri-acylated (Pam_3_CSK_4_) and di-acylated lipopeptide (Pam_2_CSK_4_) to hTLR2/1 and mTLR2/6 complex, respectively [31], [41]. It has also been demonstrated that hTLR2 possesses the di-acylated lipid binding pocket with a volume of ∼1200 Å^3^ and that hTLR1 has a single acylated lipid binding pocket with a volume of 400 Å^3^. However, the cavity volume of our hTLR10 model was 508 Å^3^. This pocket size diversity is mandatory for discrimination of structurally analogous lipopeptides. Unlike other LRR proteins, the ligand binding cavity is located in the convex region, which lies in between LRR9∼12 [31]. The domain-exchange experiments showed the importance of these regions (LRR9-12) in TLR1 and 6, which plays a crucial role in discrimination of a wide variety of ligands.

**Figure 7:**
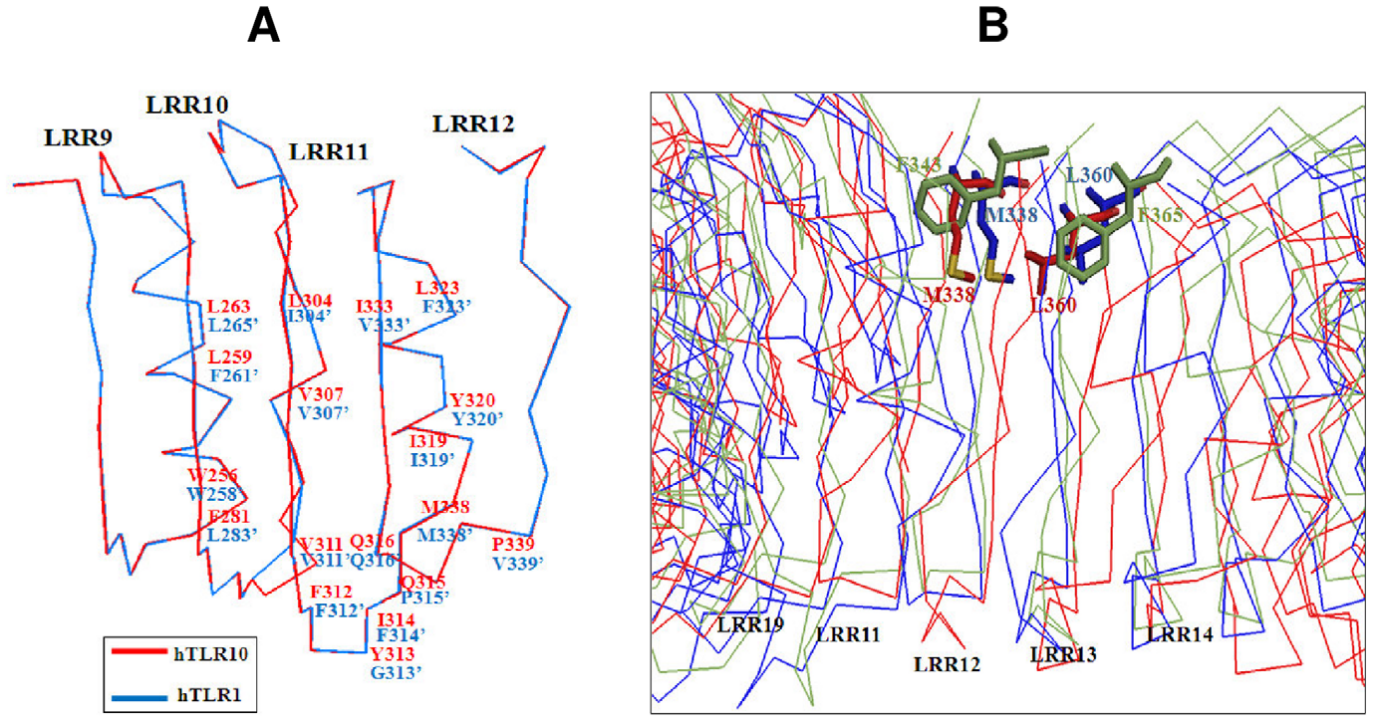
Structural comparison of ligand binding sites. (**A**) Structural comparison of the lipid-binding pockets of hTLR1 (Blue) and hTLR10 [12]. (**B**) Superimposition of the TLR6 (Green), TLR10 [12] and TLR1 (Blue): The residues responsible for the channel-blocking F343 and F365 in TLR6 are shown as green sticks and the corresponding residues M338^′^ and L360^′^ of TLR10 and M338^+^ and L360^+^ of TLR1 that are not involved in blocking are shown as red and blue sticks, respectively.

In addition to the cavity volume, we compared the hydrophobic amino acids lining the lipid binding pocket of hTLR10 (LRR9∼12) with the template hTLR1 (LRR9∼12). The results showed that out of 18 hydrophobic residues, nine were identical and the rest were replaced by other hydrophobic residues in hTLR10 (Figure 7C). Previous studies have shown the importance of M338 and L360 in TLR1, which play a crucial role in ligand recognition. Substitution of these residues in TLR1 by F343 and F365 in TLR6 results in blocking the ligand binding cavity due to the bulky side chain. However, these important residues are identical in hTLR10 (M338 and L360) and could play a significant role in recognizing a single acyl chain of lipopeptide (Figure 7D).

The results of the present study clearly demonstrate that hTLR10 might be able to accommodate single acyl chains from a tri or di-lipopeptide ligands. This provides some information that may enable identification of the ligand responsible for the activation of the hTLR10/2, hTLR10/1, and hTLR10/10 complexes. Previous studies have shown that hTLR2 can accommodate two ester bound lipid chains [31] and hTLR10 was found to possess amide bound lipid binding chains in the current study, which indicates that the tri-acylated (Pam_3_CSK_4_) lipopeptides act as ligand molecules. Additionally, the hTLR10 and hTLR1 central domain (LRR9-12) each possess a single acyl binding cavity, which indicates that neither Pam_2_CSK_4_, PamCysPamSK_4_ nor Macrophage-activating lipopeptide-2 (MALP-2) are responsible for the activation of hTLR10 homo and hTLR10/1 heterodimer complexes. So, we ruled out the possibility of the Pam_2_CSK_4_ and MALP-2 due to its nature. These ligands possess two ester bound lipid chains that run parallel to each other and it is not possible to accommodate both hTLR10 homo and hTLR10/1 heterodimer complexes due to the lack of a binding cavity. Whereas, the PamCysPamSK_4_ structure is different from the above di-acyl peptides, which have acyl chains located at the C1 and C3 atoms of glycerol (Figure S4). We have hypothesized that these two acyl chains can be accommodated by hTLR1 and hTLR10 respectively. We have tested our hypothesis by the docking experiments.

### Protein-ligand docking validation of known TLR1/2/Pam_3_CSK_4_ complexes

To evaluate our docking simulation, the crystal structure of hTLR1/2/Pam_3_CSK_4_ was downloaded from the PDB and used to conduct two different docking calculations [31]. The dominant clusters from the MOE-Dock and ASEDock docking simulations were found to have the same binding orientation when compared with the original crystal structure. The similarity between the present docked poses (ASEDock yielded bound conformations with the lowest RMSD (0.14), followed by MOE-Dock (0.38)) and the crystal structures shows that our docking protocol was able to reproduce the near native hTLR1/2/Pam_3_CSK_4_ complex. Therefore, we consider that our protocol to be trustworthy and we used it in the subsequent docking calculations.

### hTLR10 complexes and their ligand interaction

**Figure 8:**
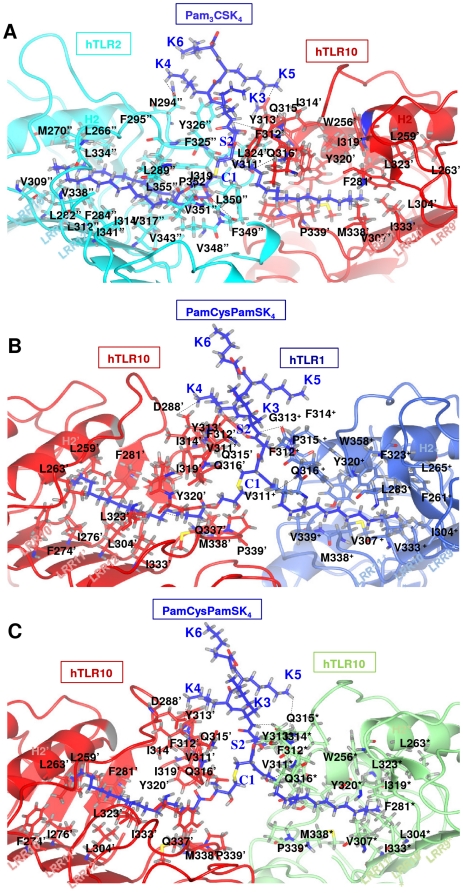
Protein-ligand docking complex. (**A**) The docked lipopeptide-binding site of the hTLR2-hTLR10 complex. The hTLR2 and hTLR10 residues involved in Pam_2_CSK_4_ binding are shown in cyan and red, respectively. Potential hydrogen bonds connecting the TLRs and the ligands are shown in broken black lines. Pam_3_CSK_4_ is shown in dark blue. (**B**) The docked lipopeptide-binding site of the hTLR10-hTLR1 complex. The hTLR10 and hTLR2 residues involved in PamCysPamSK_4_ binding are shown in red and light blue, respectively. Potential hydrogen bonds connecting the TLRs and ligands are indicated by broken black lines. PamCysPamSK_4_ is shown in dark blue. (**C**) The docked lipopeptide-binding site of hTLR10-hTLR10* complex. The hTLR10 and hTLR10* residues involved in PamCysPamSK_4_ binding are drawn in red cyan green, respectively. Potential hydrogen bonds connecting the TLRs and the ligands are shown by broken black lines. PamCysPamSK_4_ is shown in dark blue.

The hypothesized (PamCysPamSK_4_, Pam_3_CSK_4_) ligands were docked into the active site of hTLR10/1, hTLR10/2 and hTLR10/10^*^. A close up view of the Pam_3_CSK_4_ in the hTLR10/2 binding sites revealed an extensive spatial overlap of the predicted best poses, despite the variety of docking methods employed (Figure 8A). The bound conformation of the ligand present in the hTLR10/2 shows that the two ester-bound lipid chains of Pam_3_CSK_4_ are inserted in hTLR2, through a crevice formed between the LRR11 and LRR12 loops and interact with its internal hydrophobic pocket, whereas the remaining amide-bound lipid chains are inserted into hTLR10 through the gap between the LRR11 and LRR12 loops and interact with its internal hydrophobic pocket. The conserved cysteinyl group and glycerol backbone of the lipopeptide is located in the narrow opening formed, where the hTLR10 and hTLR2 pockets join. Their positions are partially fixed by five hydrogen bonds: between the backbone nitrogen of F349^′′^ and a carbonyl oxygen of the lipopeptide, between the backbone oxygen of N294^′′^ and the backbone nitrogen of K4 in the lipopeptide, between the backbone oxygen of Y313^′^ and the backbone nitrogen of K3 in the lipopeptide, between the carboxamide group of Q316^′^ and the amide oxygen in the lipopeptide and between the Q315^′^ side chain and the carbonyl backbone of K5 in the lipopeptide. The docked complex of hTLR10/1/PamCysPamSK_4_ shows a slight diverse binding mode in comparison with the TLR2/1/Pam_3_CSK_4_ and mTLR2/6/Pam_2_CSK_4_ complex.

It is important to note that the ester bound lipid chain attached to the glycerol is longer than the amide bound lipid chain. The cavity volume comparison clearly indicates that hTLR10 (518 Å^3^) is slightly higher than hTLR1 (400 Å^3^). Hence, the ester bound lipid chain cannot accommodate in hTLR1. As expected, the docking results showed that ester-bound lipid chains are recognized through the binding pocket of hTLR10 and that amide-bound chains are recognized through the binding pocket of hTLR1 (Figure 8B). There are four hydrogen bonds that are formed in the hTLR10/1 binding pocket: between the carboxylic group of D288^′^ and the NH_2_ group of K4 in the lipopeptide, between the hydroxyl group of Y320^′^ and the amide oxygen in the lipopeptide, between the carbonyl backbone of G313^+^ and the backbone nitrogen of K3 in the lipopeptide and between the carboxamide of Q316^+^ and the amide oxygen in the lipopeptide. Single apostrophes are used for the hTLR10 residues and a plus symbol for the hTLR1 monomer to differentiate them from the ligand.

In case of the hTLR10 homodimer, docking with PamCysPamSK_4_ revealed that there is a slight difference in the orientation of the ligand recognition site as well as the residual contribution in the binding pocket. The LRR11 and LRR12 loops of both hTLR10 chains are located in the center of the dimerization interface and provide key hydrophobic residues to enable recognition of the lipid chains of PamCysPamSK_4_ (Figure 8C). The hydrogen bonds present at the interface of hTLR10/10^*^ with PamCysPamSK_4_ are as follows: between the carboxylic group of Q316^′^ and the amide oxygen in the lipopeptide, between the backbone oxygen of D288^′^ and K4 in the lipopeptide, between the backbone oxygen of Y313^*^ and the backbone nitrogen of K3 in the lipopeptide, between the carboxamide of Q316^*^ and the amide oxygen in the lipopeptide and between the carboxamide of Q315^*^ and K5 in the lipopeptide. The docking studies clearly indicate that LRR9-12 present in the TLR1 family members plays a crucial role in ligand recognition, and that the hydrophobic residues present in that region are important for recognizing the acyl chain. These results are in agreement with the domain-swap experiments [31]. Furthermore, the possible ligands predicted in this study are in agreement with those of a previous study conducted by Hasan et al., who reported that the ligands for TLR1 family such as tri- and di-acylated lipopeptides might be the same for hTLR10 [12]. Previous studies have shown that NF-κB, ENA-78 and other gene promoters are activated by an unknown ligand through the TLR10 pathway [42]. Based on our current study, PamCysPamSK_4_ and Pam_3_CSK_4_ might be possible ligands for the activation of TLR10 signaling pathways.

Taken together, we elucidated the 3D structure of hTLR10 to show its homo and heterodimerization with hTLR1 and hTLR2. Based on these models, we also suggest three possible receptor dimerization schemes that require different minimum ligand sizes. These complexes provided the specific active sites, which enabled us to predict the possible ligands necessary for the TLR10 activation. We also proposed that PamCysPamSK_4_ and Pam_3_CSK_4_ are the possible ligand molecules for the TLR10 pathway. Our models provide a structural framework for interpreting experimental data and allow predictions of the TLR signal transduction process. The presented modeling approach can be extended to other repetitive protein domains.

## Supporting Information

Figure S1 The backbone superimposition of hTLR10 with hTLR2 and hTLR1 are shown as red, cyan and blue, respectively.
(1.75 MB TIF)

Figure S2 The Ramachandran plot of refined hTLR10, 2 and 1.
(1.85 MB TIF)

Figure S3 Electrostatic potential on the molecular surface of hTLR10, 2 and 1. The LRR9-17 patches of hTLR10, 2 and 1 are circled. Red and blue indicates negative and positive potential, respectively. The surface potential was calculated and displayed using the PyMOL ABPS tool.
(2.64 MB TIF)

Figure S4 Chemical structure of lipopeptides used for docking studies.
(0.76 MB TIF)
