# Supplementary figures and images for: Molecular Modeling-Based Evaluation of hTLR10 and Identification of Potential Ligands in Toll-Like Receptor Signaling
Source: PLoS One. 2010 Sep 16;5(9):e12713. doi: 10.1371/journal.pone.0012713 (PMC2943521; doi:10.1371/journal.pone.0012713)

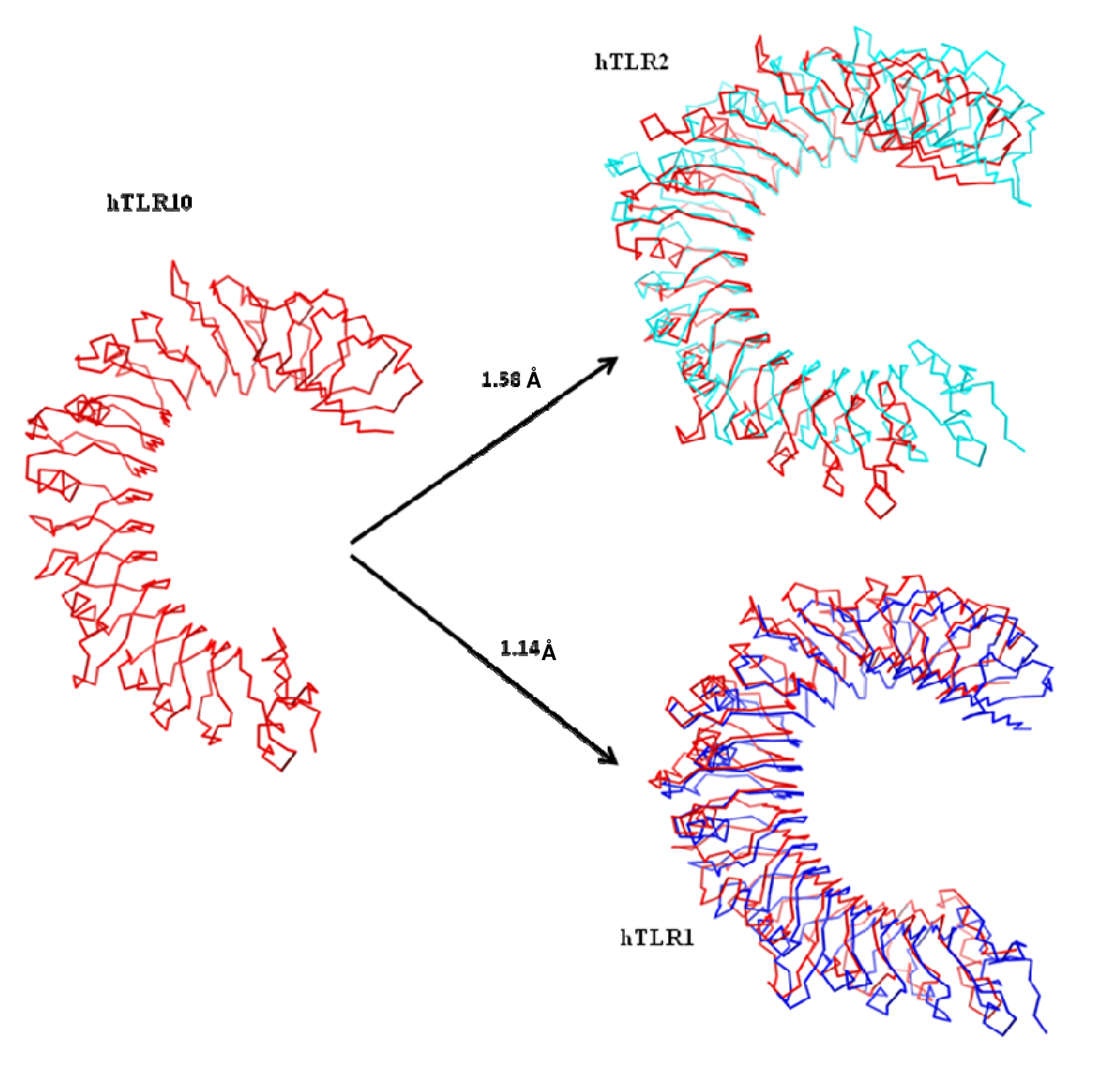

Supplement: Figure S1 — The backbone superimposition of hTLR10 with hTLR2 and hTLR1 are shown as red, cyan and blue, respectively. (1.75 MB TIF) [file pone.0012713.s001.tif]

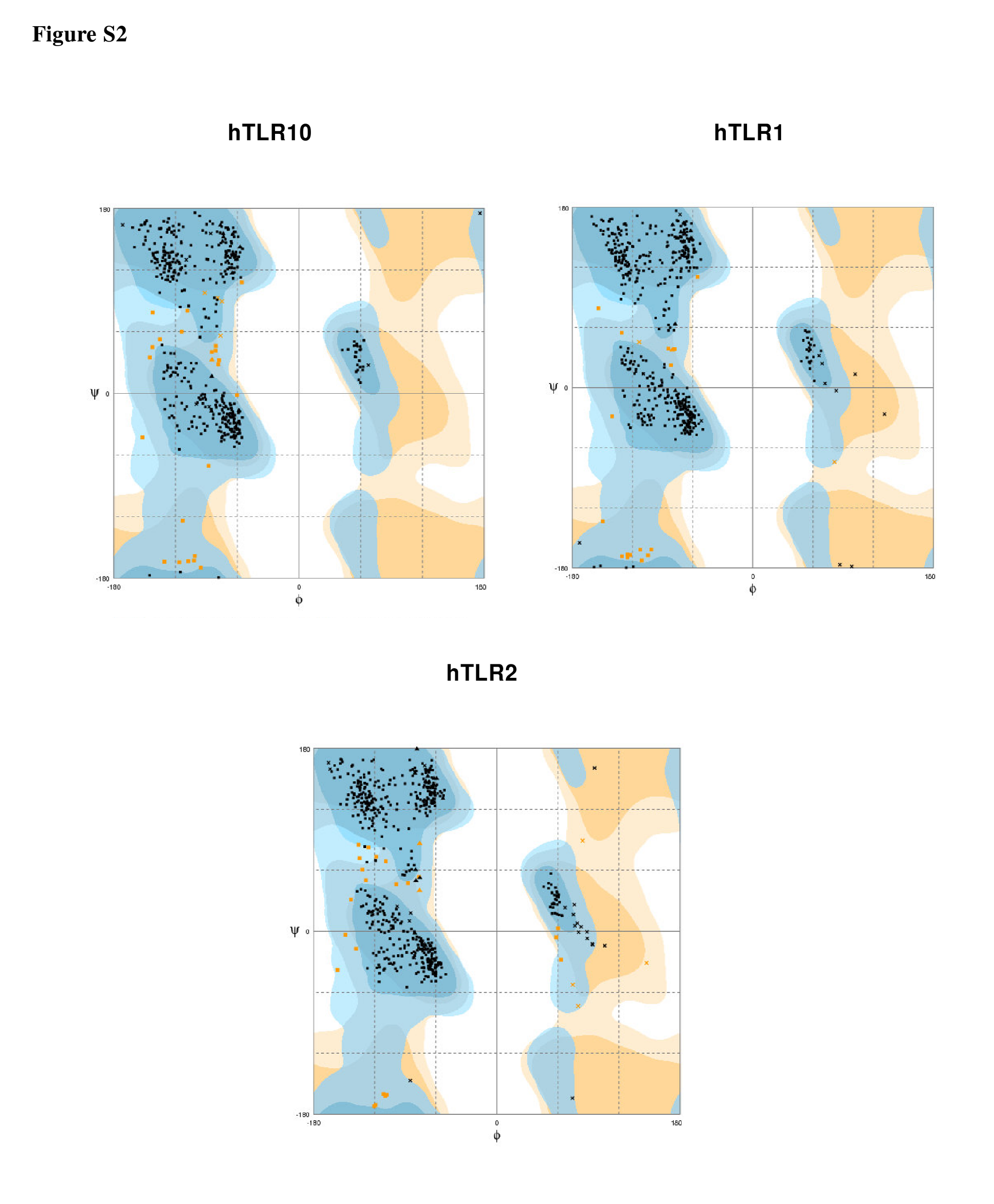

Supplement: Figure S2 — The Ramachandran plot of refined hTLR10, 2 and 1. (1.85 MB TIF) [file pone.0012713.s002.tif]

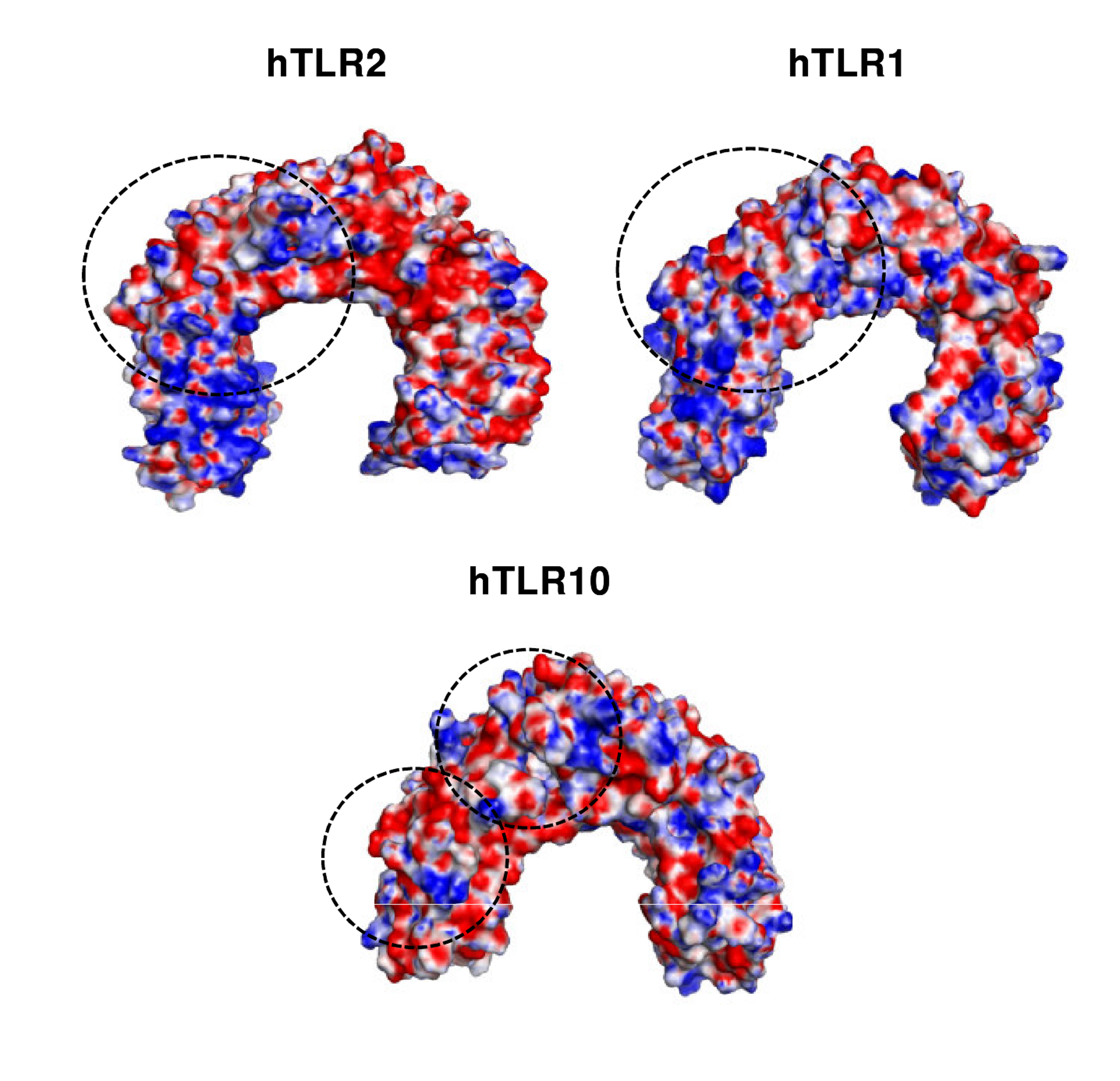

Supplement: Figure S3 — Electrostatic potential on the molecular surface of hTLR10, 2 and 1. The LRR9-17 patches of hTLR10, 2 and 1 are circled. Red and blue indicates negative and positive potential, respectively. The surface potential was calculated and displayed using the PyMOL ABPS tool. (2.64 MB TIF) [file pone.0012713.s003.tif]

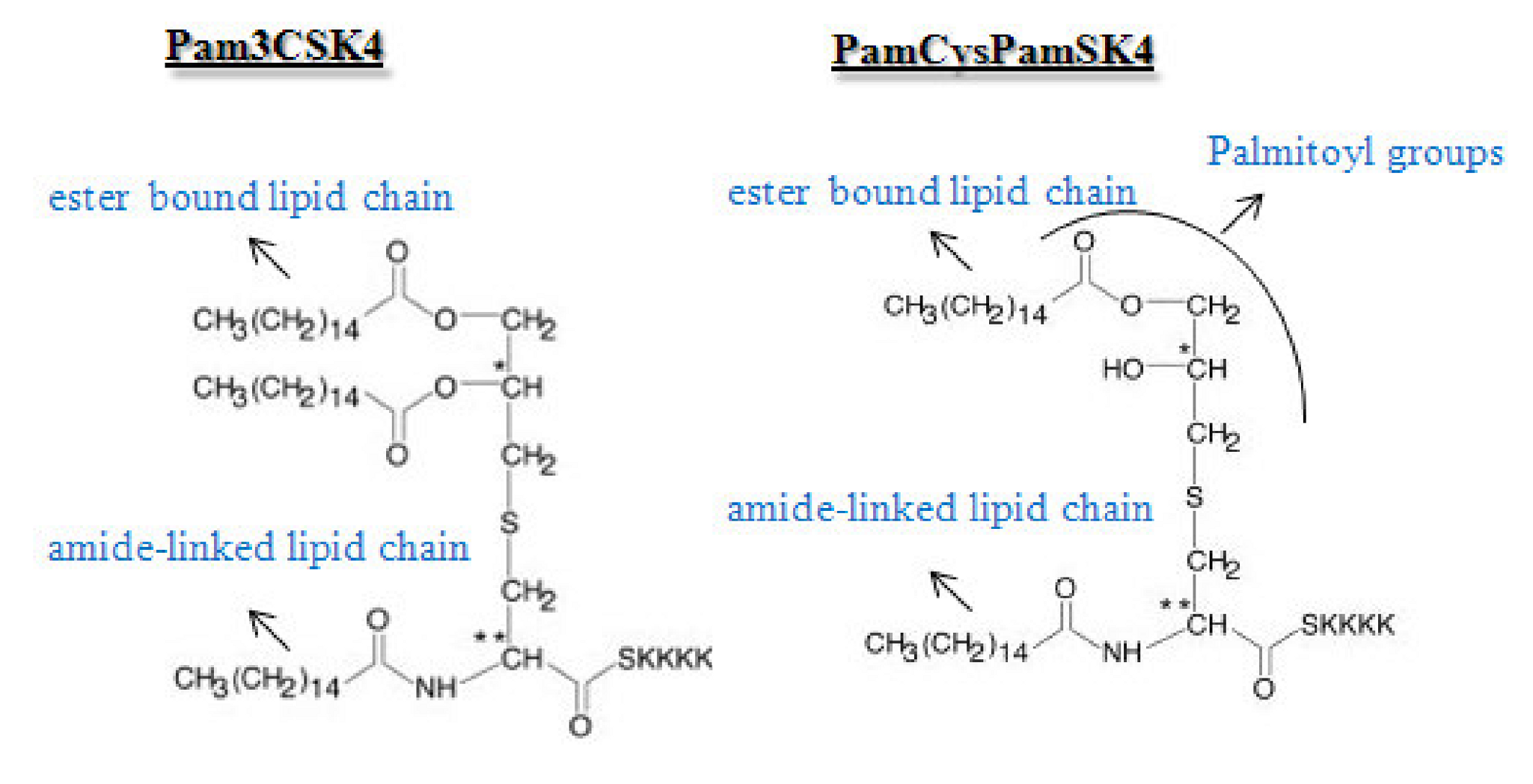

Supplement: Figure S4 — Chemical structure of lipopeptides used for docking studies. (0.76 MB TIF) [file pone.0012713.s004.tif]
